# Adaptive Immunity Regulation and Cerebral Ischemia

**DOI:** 10.3389/fimmu.2020.00689

**Published:** 2020-05-12

**Authors:** Xingping Qin, Farhana Akter, Lingxia Qin, Jing Cheng, Mei Guo, Shun Yao, Zhihong Jian, Renzhong Liu, Songlin Wu

**Affiliations:** ^1^Department of Neurosurgery, Renmin Hospital of Wuhan University, Wuhan, China; ^2^Massachusetts General Hospital Cancer Center and Harvard Medical School, Boston, MA, United States; ^3^Faculty of Arts and Sciences, Harvard University, Cambridge, MA, United States; ^4^Department of Neurology, Renmin Hospital of Wuhan University, Wuhan, China; ^5^Department of Neurosurgery, Center for Pituitary Surgery, The First Affiliated Hospital, Sun Yat-sen University, Guangzhou, China; ^6^Department of Neurosurgery, Center for Skull Base and Pituitary Surgery, Brigham and Women's Hospital and Harvard Medical School, Boston, MA, United States; ^7^Department of Geriatrics, Renmin Hospital of Wuhan University, Wuhan, China

**Keywords:** cerebral ischemia, stroke, immune response, innate immunity, adaptive immunity

## Abstract

Stroke is a disease that occurs due to a sudden interruption of the blood supply to the brain. It is a leading cause of death and disability worldwide. It is well-known that the immune system drives brain injury following an episode of ischemic stroke. The innate system and the adaptive system play distinct but synergistic roles following ischemia. The innate system can be activated by damage-associated molecular patterns (DAMPs), which are released from cells in the ischemic region. Damaged cells also release various other mediators that serve to increase inflammation and compromise the integrity of the blood–brain barrier (BBB). Within 24 h of an ischemic insult, the adaptive immune system is activated. This involves T cell and B cell-mediated inflammatory and humoral effects. These cells also stimulate the release of various interleukins and cytokines, which can modulate the inflammatory response. The adaptive immune system has been shown to contribute to a state of immunodepression following an ischemic episode, and this can increase the risk of infections. However, this phenomenon is equally important in preventing autoimmunity of the body to brain antigens that are released into the peripheral system as a result of BBB compromise. In this review, we highlight the key components of the adaptive immune system that are activated following cerebral ischemia.

## Introduction

Stroke occurs due to a compromise of the blood supply to a particular region of the brain leading to permanent neurological deficits (1) such as weakness, sensory deficits, visual field defects, and aphasia (2). Approximately 85% of all cases of stroke are of the ischemic type, and it is the third leading cause of death in the United States (3).

**Figure 1:**
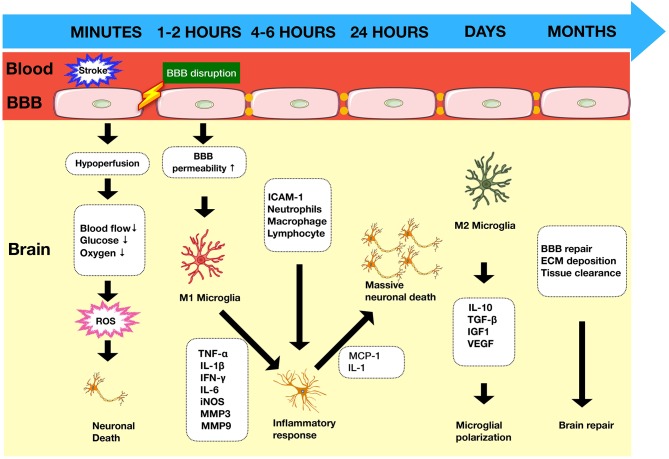
Timeline of immune response in ischemic stroke. Ischemic stroke begins with a cascade of events as a result of arterial occlusion leading to hypoxia accompanied by ROS production within minutes and glucose deprivation. The integrity of the BBB is compromised leading to increased permeability within 1–2 h, allowing infiltration of circulatory cells which contribute to the inflammatory process and exacerbate neuronal death. BBB, blood–brain barrier; ROS, reactive oxygen species; MMPs, matrix metalloproteinases; DAMPs, damage-associated molecular patterns; ECM, extracellular matrix.

**Figure 2:**
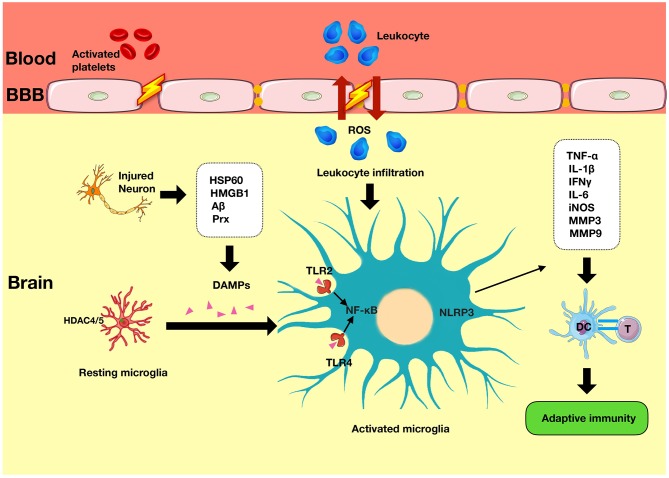
Innate immunity in ischemic stroke. Immediately following an episode of stroke, the innate immune system is activated. It is characterized by the presence of various mediators such as DAMPs that have been released from the injured neurons and which induce the secondary activation of microglia *via* TLRs signaling and NLRP3 activity. TLR, Toll-like receptors; NLRP3, nod-like receptor pyrin domain-containing 3; APC, antigen-presenting cell; DAMPs, damage-associated molecular patterns.

The ischemic cascade is initiated following the onset of stroke. It is characterized by a loss of adenosine triphosphate (ATP) leading to ion pump failure, accumulation of intracellular calcium, and glutamate-induced excitotoxicity of cells (4). In addition to the central neuronal response, there is activation of immune responses in the injured tissue. The cells most sensitive to ischemic damage in the brain are neurons (5). Dying neuronal and non-neuronal cells release damage associated molecular patterns (DAMPs) such as high-mobility group box 1 (HMGB1) and heat shock proteins (HSPs) (Figure 1). These activate resident cells such as microglia (6), which alongside other pro-inflammatory mediators contribute to disruption of the blood–brain barrier (BBB). Once the BBB has been compromised, there is a prolonged flux of systemic inflammatory cells such as monocytes, neutrophils, and T cells into the injured area, which further exacerbates the injury (Figure 2).

Activation of T and B cells is a key component of the adaptive immune system and occurs within 24 h following injury (7). T cell activation occurs following recognition of T cells by the T cell receptor and engagement of costimulatory molecules such as cluster of differentiation (CD) 28 with B7 or those from the tumor necrosis factor (TNF) receptor family such as CD137 (TNFRSF9, 4-1BB) (8). The adaptive system appears to play a beneficial role following cerebral ischemia. However, there is also evidence that activation of the adaptive system may exacerbate the ischemic injury and contribute to systemic immune suppression, leading to increased susceptibility to infections (9). A recent study showed that CD137 costimulation is associated with increased systemic inflammation following cerebral ischemia and may exert deleterious effects (10).

Although advances in the field have been made and are continuing to evolve, our understanding of the process is by far from complete. In this review, we highlight the key components of the adaptive immune system in cerebral ischemia and discuss potential therapeutic targets.

## Adaptive Immunity in Cerebral Ischemia

**Figure 3:**
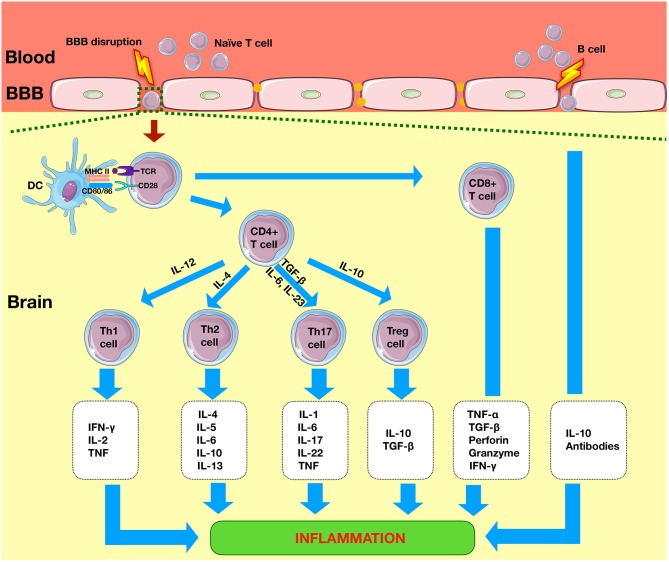
Adaptive immunity in ischemic stroke. Within 1 day of the ischemic event, there is infiltration of system cells such as CD4+ T and CD8+ T cells. CD4 cell cells differentiate into Th1, Th2, Th17, or Tregs to produce pro-inflammatory or anti-inflammatory effects. CD8+ T cells lead to neuronal death by release of perforin and granzyme. B cells produce anti-inflammatory effects *via* the release of interleukins such as IL-10. TLR, Toll-like receptors; NLRP3, nod-like receptor pyrin domain-containing 3; APC, antigen-presenting cell; CD4+, cluster of differentiation 4+; Th, T helper; Treg, regulatory T cell.

The key cells involved in the adaptive immune system are T cells and B cells. T cells are divided into CD8^+^ cytotoxic T cells and CD4+ T helper (Th) cells. Most T cells are of the alpha beta (αβ) type, and the remaining are of the gamma delta (γδ) type. The presence of these cells in the healthy brain is limited and regulated by the intact BBB (11); however, following an episode of ischemia, they rapidly infiltrate the diseased brain (Figure 3) (12).

### Timing of the Components of the Adaptive System

The first cells of the adaptive immune system to migrate to the ischemic region are the CD8+ cytotoxic cells, which can be seen as early as a few hours following stroke (12) and are usually abundant between 1 and 7 days post injury (13).

Between 1 and 2 days post ischemia, T cells have been observed in the subpial region, and by day 7, they can be seen in the edges of the ischemic region (14). T cells reduce in number by day 14 (14) but have been observed in the peri-infarct area up to 1 month following injury (15, 16). T cell activation markers CD44 and CD25 and pro-inflammatory cytokines are also present in the ischemic region (15). Infiltration of CD4+ cells has been observed within 24 h after the onset of ischemia (17). Regulatory T cells (Tregs) have been observed a few days after the onset of ischemia and can persist for more than 30 days (18, 19). Interestingly, the time-dependent increase of T cells can differ by sex, with higher levels of CD8+ T cells and Tregs observed at day 15 in aged male mice compared to females despite a similar pattern of ischemic brain injury (20).

## Cytotoxic CD8+ T Cells

CD8+ lymphocytes cells are widely found in the ischemic penumbra (15). CD8^+^ T cells may play deleterious roles in the ischemic infarct, with evidence of reduced infarct size and improved neurological outcome following induced cerebral ischemia in CD8+ knockout models (21) and antibody depletion models (22). CD8+ T cell damage may lead to direct neuronal cytotoxicity *via* a number of pathways such as the Fas ligand (FasL) pathway, with evidence of reduced neuronal cell death following use of FasL and 3-phosphoinositide-dependent protein kinase 1 (PDPK) inhibitors (23). CD8+ T cell-induced neuronal damage can also occur *via* humoral pathways by releasing inflammatory mediators such as interferon gamma (IFN-γ) (24). It is unclear whether CD8+ T cells are a major source of IFN-γ, and there is conflicting evidence of whether IFN-γ plays a pivotal (21) or a minimal role in the evolution of the infarct under normal conditions (25). CD8+ cells have been shown to release various other cytokines such as interleukin 16 (IL-16), which have been found in the ischemic penumbra. IL-16 serves to recruit and activate immune cells and can lead to blood vessel and BBB damage. They have been found to accumulate in the necrotic lesion and can reach maximum levels 3–4 days after ischemia (26). In addition to releasing pro-inflammatory cytokines, cytotoxic CD8+ T cells can also mediate their actions *via* direct cytolytic pathways by releasing cytotoxic proteins such as granzymes and perforin (22). Cerebral ischemia is associated with white matter injury.

A major pathobiological change seen following white matter brain injury is demyelination. Interestingly, demyelination is closely related to T cell-mediated changes. It has recently been shown that cytotoxic CD8+ T cells may worsen white matter injury and demyelination in cerebral ischemia. Given that CD8+ differentiation can be affected by IL-2 homeostasis; one group showed that IL-2 monoclonal antibody can preserve white matter integrity following experimental ischemia and therefore may be a useful therapeutic target (27).

### γδ T Cells

Gamma delta (γδ) T cells represent a small subset of T cells. The majority of these cells are activated in a major histocompatibility complex (MHC)-independent manner, in contrast to the classic MHC-restricted αβ T cells. γδ T cells play a role in both the innate immune system and the adaptive immune system following an episode of stroke. They have been detected in infarcts at 6 h post ischemia. They can secrete IL-17 and mediate the infiltration of pro-inflammatory cytokines (28, 29). IL-17 works synergistically with TNF-α to induce neutrophil infiltration *via* chemokines such as chemokine (C-X-C motif) ligand 1 (CXCL-1). Blocking IL-17 or CXCL-1 has been shown to reduce infarct size (30). However, IL-17-mediated effects are short lived and therefore may not be responsible for the prolonged inflammation seen after ischemia.

### CD3^+^CD4^−^CD8^−^ T Cells (Double-Negative T Cells)

Studies investigating the role of T cells in ischemic stroke have predominantly focused on infiltrating CD3^+^CD8^+^ and CD3^+^CD4^+^ T cells (31). However, the role of double-negative T cells (DNTs), which account for 1–5% of the total T cell population, has seldom been investigated (32). This is perhaps because the function of DNTS in the normal brain is not fully known. All T cells begin as a double-negative cell and subsequently rearrange into the common CD4+/CD8+ forms. In a mouse model of cerebral ischemia, it was demonstrated that DNTs infiltrate the brain 1–3 days after ischemia and are located near activated microglia. DNT-derived TNF-α was shown to contribute to activation of microglia *via* the FasL pathway, and TNF-α expression in DNTs was regulated by T cell protein tyrosine phosphatase (PTPN2). Targeting the FasL/PTPN2/TNF-α signaling pathway may therefore be an attractive option in treating ischemic stroke (33). However, the role of DNTs in the ischemic brain is not fully understood, and certainly their role in both the innate and the adaptive systems including their interactions with other cells and cytokine release profile need to be further explored.

## T Helper Cells

CD4^+^ T cells are divided into conventional Th cells and Tregs. Th cells play a crucial role in the adaptive immune system. There are two effector CD4+ Th cell responses that can be induced, and these are designated Th1 and Th2. The Th1 response is characterized by the production of IFN-γ, which has been shown to create a delayed immune response to stroke. Blocking the IFN-γ signaling pathway reduces inflammatory chemokine IFN-inducible protein 10 (IP-10) expression and decreases neurodegeneration (34) following middle cerebral artery occlusion (MCAO). Levels of IFN-γ and IP-10 are also reduced following CD4 T cell depletion 3 days after stroke onset, and this is associated with improved behavioral outcomes (35). However, other studies have shown that mice deficient in IFN-γ developed infarcts similar to control (25).

The presence of Th2 cells can lead to a humoral immune response characterized by the release of various ILs such as IL-10. IL-10 has been shown to play a central immunomodulatory role and can reduce infarct volume. In the MCAO model, IL-10^−/−^ C57BL/6 mice were shown to have 30% larger infarct volumes compared with wild-type mice 24 h following a stroke (36). IL-10 mediates its anti-inflammatory effect by downregulating various pathways such as the nuclear factor kappa-light-chain enhancer of activated B cells (NF-κB). One study demonstrated improved BBB integrity in the cerebral ischemia–reperfusion model using hydrogen sulfide donors, and this was accompanied by enhanced IL-10 expression and reduced NF-κB nuclear translocation (37). The extent of the impact of Th2 cytokines ischemic sequelae in mice subjected to MCAO is however unclear (38).

Various pro-inflammatory mediators are elevated in CD4+ T cells following ischemia. A recent study showed increased expression of soluble CD137 on CD4+ T cells in peripheral blood of patients following an episode of stroke compared to controls (39) and may therefore be a potential therapeutic target. Various novel immune modulatory targets of cerebral ischemia have been recently investigated. One study investigated the role of acetyl coenzyme A carboxylase 1 (ACC1), an enzyme that mediates fatty acid synthesis and CD4^+^ T cell-associated inflammation. Following transient MCAO, there was evidence of increased fatty acid synthesis and expression of ACC1 in CD4+ T cells. Inhibition of ACC1 depletion was associated with reduced neuro-inflammation, preservation of peripheral Tregs/Th17 cells and improved neurological outcome following ischemia (40).

### Regulatory T Cells

Regulatory T cells (Tregs) are a type of CD4+ cells that can suppress the proliferation of CD4+ and CD8+ T cells and maintain self-tolerance (41). The most well-understood of the naturally occurring Tregs are those that express CD4, CD25, and forkhead/winged-helix transcription factor box P3 (FOXP3). However, these markers are not specific for Tregs and are in fact T-cell activation markers (41). Tregs have been found in both normal healthy brains and also following an ischemic episode up to 30 days post injury (18).

Whether Tregs are a core component of the adaptive immune system or the innate immune system is the subject of much controversy, Liesz et al. showed that endogenous T_regs_ are protective in later stages of stroke (42) and that their beneficial functions depend on IL-10 (28). However, a recent study suggests that T_regs_ have an early detrimental role by inducing dysfunction of the cerebral microcirculation (37) and are therefore more important for the innate system. Tregs can induce microvascular dysfunction *via* the lymphocyte function-associated antigen 1/intercellular adhesion molecule (LFA-1/ICAM-1) pathway, and ablation of Tregs can improve cerebral reperfusion in stroke (43).

Many studies however show that adoptive transfer of Tregs can improve outcome following stroke (18), with worse neurological outcomes and larger infarcts seen in ischemic mice with Treg depletion (42). A number of different mechanisms of Treg-mediated neuroprotection exist. These include Treg-induced increase in IL-16 (44) and reduction in inflammatory mediators such as NF-κB (45). Tregs can also suppress matrix metalloproteinase 9 (MMP9) production in neutrophils and thereby prevent breakdown of the BBB (46, 47). Despite Tregs being an attractive target in ischemic stroke, their low frequency limits their clinical use. Effective methods to expand Tregs *in vivo* are therefore desirable.

The IL-2/IL-2 antibody complex (IL-2/IL-2Ab) has been shown to increase the number of Tregs and promote the expression of CD39 and CD73 in expanded Tregs in experimental ischemia models. This is associated with a reduced infarct size, improved neurological outcome. Tregs can be expanded using IL-33 (48), which can improve outcomes following ischemia by reducing IFN-γ^+^ T cells and increasing Foxp3^+^ T cells in the spleen (49). However, the beneficial effects of IL-33 may be mitigated by an increased rate of respiratory infections (50). Brain Tregs express neural cell-specific genes such as the serotonin receptor (Htr7) and can be modulated with serotonin or selective serotonin reuptake inhibitors (SSRIs) to reduce neurological symptoms (51). Another method of Treg expansion is the use of poly (ADP-ribose) polymerase-1 (PARP-1) inhibitors, which has been shown to increase the proportion of Tregs in peripheral blood mononuclear cells (PBMCs), reduce pro-inflammatory cytokines (IFN-γ, TNF-α, and IL-17), and increase inflammatory cytokines [IL-4, IL-10, and transforming growth factor β1 (TGF-β1)] (52). The signaling protein sirtuin is known to reduce T cell activation and is a negative regulator of Treg function (53). Cerebral ischemia is associated with increased expression of sirtuin in infiltrating Tregs, and this is mediated by the transcription factor, hypoxia-inducible factor 1-alpha (HIF-1α) (54), which can be modulated to expand Treg numbers.

### T Helper 17 Cells

There are various Th cells which are closely related to Tregs and are involved in cerebral ischemia. These include Th17 cells, which produce IL-17. Th17 cells mediate the recruitment of neutrophils and macrophages to damaged tissues. These cells are thought to be the preferential producers of iIL-17A, IL-17F, IL-21, and IL-22. IL-17 can also be produced by other cells such as neuroglial cells (11) and γδ T lymphocytes (55).

In a study of patients with ischemic stroke, IL-17A was found to be significantly expressed at day 7 following a stroke. It continues to be present up to day 28, although significantly less than day 7. It must be noted however that patients were taking various anti-inflammatory medications from day 7 onward, which may have affected the results. Levels of Tregs followed an opposite pattern and were markedly reduced in patients at day 7 but increased after day 28. The interaction between IL-17A and Tregs and its significance are not fully understood (56).

The mechanism of IL-17-induced damage following ischemia is not fully understood; however, one study demonstrated evidence of increased activation of the calpain-transient receptor potential canonical (subtype) 6 (TRPC6) signaling pathway, which is known to be involved in cerebral ischemia (47). TRPC6 is a cation channel that protects neurons from excitotoxicity and ischemic damage. Cerebral ischemia is associated with intracellular calcium overload leading to activation of calpain, which hydrolyzes TRPC6. Suppression of TRPC6 degradation may therefore be useful in cerebral ischemia to preserve neurons. However, there are many other mechanisms of calpain-mediated downregulation of TRPC6 contributing to ischemic brain injury and therefore without accounting for other factors, it is difficult to measure the significance of IL-17-mediated calpain damage (56).

## B Cells

B Cells are the key players in humoral immunity. Upon activation, B cells produce antibodies, which recognizes specific antigens. The role of B cells in cerebral ischemia is inconclusive. Some found a beneficial role of B cells (57, 58). Whereas, others found no impact on infarct volume and functional outcome (21, 31, 59).

The beneficial effects of B cells include reduced infarct volume compared to the control group (60). B cells can also limit the production of cytokines such as IFN-γ and TNF-α and infiltration of inflammatory T cells (57). Cerebral ischemia is associated with splenic atrophy, which is associated with a reduction of inflammatory cytokines, T cells and B cells. The profound loss of B cell limits the ability of the humoral immune system to provide protection, and this contributes to systemic immunosuppression (61). Splenectomy, on the other hand, can reduce the pool of immune cells and rats splenectomized 2 weeks before experimental ischemia show reduced infarction volume in the brain (62).

A number of studies have shown that B cells may not influence infarct size and outcome in ischemic models. Mice homozygous for the *Rag*1^−/−^ mutation which produce no mature T cells or B cells did not have reduced infarct size following ischemia compared to wild type. Interestingly, *Rag1*^−/−^ mice supplemented with T cells developed significant ischemic brain damage (63).

## Natural Killer Cells in the Adaptive System

Natural killer (NK) cells are key players of the innate immune system; however, they also have attributes of the adaptive system. Following an ischemic episode, there is an increased amount of NK cells found in brain tissues (64) within 3 h, with peak levels between days 3 and 5 (64). NK cells modulate adaptive immunity *via* cytokine production such as IFN-γ, which can induce neuronal necrosis in cerebral ischemia. NK cells can also modulate the adaptive immune response *via* the perforin-granzyme-mediated cytolytic pathway. Granzymes and perforin are stored in the cytoplasmic granules of NK cells and when released can augment local inflammation (64). Additional evidence of NK cells being a part of the adaptive immune system in cerebral ischemia are from an observed increase of circulating NK cells in patients with post-stroke infections suggesting that they have a role in post-stroke immunodepression (64). NK cells are therefore an attractive target in cerebral ischemia. However, it is not clear how modulating NK cells would affect infarct evolution or immunodepression. Furthermore, NK cells are well-known players of the innate immune system, and therefore the timing of NK cell modulation may play a crucial role in its effect on the ischemic infarct.

## Immunological Tolerance

Following an episode of stroke, there is a breakdown of the BBB, which allows antigens from the CNS to migrate into the systemic circulation to be recognized by the peripheral immune system and modulate an inflammatory response. Many researchers have exploited this mechanism to induce immunological tolerance to a specific antigen such as MBP. MBP is a protein which increases in the early period after acute ischemic stroke (65). Using an induced oral tolerance to antigen model, it was shown that cell-mediated Th1 immune responses are involved following an episode of stroke. In an experimental MCAO model, rats were fed MBP 3 h before the procedure. This is a common model to induce immunological tolerance to a specific antigen by feeding of that antigen, which can lead to clonal depletion of antigen-reactive T cells and cause active tolerance. Upon re-stimulation with the antigen, the T cells in the animal which has been tolerized can secrete cytokines such as TGF-β1, and this can then suppress Th1 immune response and lead to deviation toward a humoral Th2 response. This leads to bystander immunosuppression, where the activation of T cells led to non-specific effects despite being activated in an antigen-specific manner (66, 67). Immunological tolerance to antigens such as MBP could therefore decrease cerebral infarction *via* the bystander suppression phenomena. Indeed, in the MCAO model, rats that were fed MBP had reduced infarct size up to 4 days post procedure compared to control. Immunohistochemistry revealed increased levels of TGF-β1 production by T cells in the brain, suggesting that modulation of the antigen-specific modulation can decrease infarct size (68). MCAO however is also associated with an autoimmune response to the brain MBP. One month after MCAO, lymphocytes from spleens of MBP tolerized animals show a regulatory response (Treg) toward MBP compared to control. Rats which demonstrated an inflammatory response (Th1) had worse neurological outcome (69). However, the benefit of this form of therapy does not extend beyond 3 months after MCAO, which could be due to induction of mucosal tolerance to MBP only serving to delay recovery. Mucosal administration of antigen may also lead to detrimental autoimmunity (70).

E-selectin (CD62E) is a cell adhesion molecule, which is activated following an inflammatory insult that can guide tolerized Tregs to blood vessels where they can release cytokines to inhibit the development of ischemic stroke (71). E-selectin is distributed in the vascular endothelium and can serve as an antigenic target for Treg guidance to vessel segments. E-selectin has been shown to reduce infarct size in the MCAO stroke model. Rats tolerized to nasally administered E-selectin had a reduction in brain infarct volume compared with controls. A similar effect was seen following adoptive transfer of splenocytes from E-selectin-tolerized donors (72). Th1 cells are generally regarded as mediators of delayed-type hypersensitivity (DTH) reactions. E-selectin-specific DTH responses were significantly suppressed in E-selectin-tolerized animals. Suppression of the Th1-mediated DTH reaction to E-selectin provides evidence for the generation of antigen-specific Tregs in rats tolerized to E-selectin. These findings demonstrate cell-mediated immunomodulation as the basis for the observed cytoprotection in this MCAO stroke model (72).

A recent study demonstrates that cerebral ischemia induces varied neuroantigen-mediated T cell responses, which can exacerbate the injury. Brain ischemia induces diversified neuroantigen-specific T cell responses that exacerbate brain injury. This was confirmed using adoptive transfer of neuroantigen-experienced MOG_35−55_ (myelin oligodendrocyte glycoprotein) epitope (2D2) which worsened brain injury by inducing Th1/Th17 responses (73).

## Post-Stroke Immunodepression

The adaptive immune system plays a significant role in post-stroke immunodepression and susceptibility to infection. During this period, there is evidence of a shift from cell-mediated inflammatory Th1 type response to a humoral-mediated anti-inflammatory Th2 type response. This occurs to protect the brain from further damage. However, this is in detriment to the body's systemic immune system, which is suppressed and therefore there is increased risk of developing systemic infections (74). There is increased Th2 cytokine production and reduction of T, B, and NK lymphocytes, TNF-α, and IFN-γ. Administration of IFN-γ at day 1 after stroke however can reduce the development of infection (75).

Three days following induced cerebral ischemia in mice, there is evidence of increased incidence of infections such as pneumonia, a major cause of mortality. Stroke-induced immunosuppression directly affects pulmonary immunity and leads to an increased risk of bacterial infection. Following an episode of stroke, there is increased percentage of alveolar macrophages and neutrophils and reduced amount of CD4^+^ T cells, CD8^+^ T cells, B cells, NK cells, and eosinophils in the lungs (76). CD147 or extracellular matrix metalloproteinase inducer (EMMPRIN) is a transmembrane glycoprotein and a member of the immunoglobulin superfamily, which is upregulated in the lungs following stroke. Inhibition of CD147 leads to reduced pulmonary edema, and this is associated with IL-17A expression in lung γδ T cells. It may therefore be a promising therapeutic adjunct in stroke-associated pneumonia (77).

**Figure 4:**
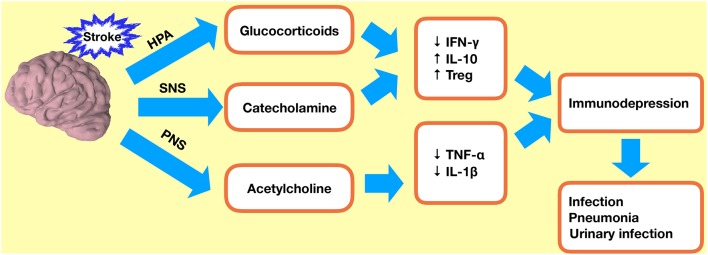
Immunodepression after ischemic stroke. Stroke-induced immunodepression leads to an increased incidence of infections. HPA, hypothalamic–pituitary–adrenal axis; SNS, sympathetic nervous system; PNS, parasympathetic nervous system; Treg, regulatory T cell.

Bacterial infections are also thought to be increased in response to activation of the sympathetic nervous system (SNS) and the hypothalamic–pituitary–adrenal (HPA) axis (Figure 4). Noradrenaline (NA), the main neurotransmitter of the SNS, can regulate immune cell development (78). Plasma levels of NA are elevated following ischemia, and this is associated with a higher incidence of infections (79). Studies have shown that reduced IFN-γ response and associated infections following ischemia can be prevented by targeted inhibition of SNS signaling (75, 80, 81). It is not possible to inhibit the entire SNS or the HPA axis in patients, and therefore the extent to which these pathways are clinically relevant needs to be further elucidated in animal models.

## Limitations

Despite significant advances in the field, the role of adaptive immunity in cerebral ischemia is far from being well-understood. The greatest concern is the observed opposing effects of cells and mediators seen in different experimental models, limiting their applicability to humans. Although many groups use the MCAO method to induce ischemia, the wide variations in inflammatory responses between rodents and humans remain an issue. Furthermore, the presence of other comorbidities which may have an effect on the inflammatory system cannot be replicated in rodents, and this itself raises questions regarding the generalization of the data. Another key factor is the vast interactions between immune cells which cannot be replicated in a single study. This has implications for application to patients, as there needs to be a fine balance between inducing the beneficial aspects of the immune system while preventing deleterious effects. The greatest challenge therefore is identifying and modulating the most significant immune target in cerebral ischemia.

## Conclusions

The brain is an immune-privileged organ due to the presence of the BBB. However, during an episode of ischemia, the integrity of this BBB is compromised. Cells of the adaptive immune system play varied roles within an ischemic episode. There is clear evidence of CD8+ cells migrating into the infarcted region following ischemia; this serves to increase inflammation with release of numerous cytokines and ILs. However, CD4+ cells and Tregs are also increased in the infarcted region and can play a protective role. Ischemic stroke is associated with post-stroke immunodepression, and this is known to be associated with increased incidence of infection. However, it is also required to prevent stroke-related autoimmunity. Following ischemia, CNS antigens enter the peripheral circulation and leukocytes to enter the brain. This can increase the likelihood of developing an autoimmune response to the brain after a stroke. Post-stroke immunodepression is therefore an adaptive response to acute cerebral ischemia and is required to reduce the likelihood of developing autoimmunity to CNS antigens.

## Author Contributions

XQ, RL, and SW designed the paper and recommended a structure for the review. XQ and FA wrote the initial draft and revised the manuscript. Figures and submission were prepared by LQ, JC, and MG. SY, ZJ, and SW helped to revise the manuscript.

## Conflict of Interest

The authors declare that the research was conducted in the absence of any commercial or financial relationships that could be construed as a potential conflict of interest.
